# A nomogram prediction model for early death in patients with persistent pulmonary hypertension of the newborn

**DOI:** 10.3389/fcvm.2022.1077339

**Published:** 2022-12-22

**Authors:** Chuyang Lin, Jiao Mi, Yinyue Zhang, Sichen Duan, Jinlin Wu, Yifei Li

**Affiliations:** Key Laboratory of Birth Defects and Related Diseases of Women and Children of MOE, Department of Pediatrics, West China Second University Hospital, Sichuan University, Chengdu, Sichuan, China

**Keywords:** persistent pulmonary hypertension of the newborn, neonatal death, nomogram prediction, risk factors, clinical outcome

## Abstract

**Background:**

Persistent pulmonary hypertension of the newborn (PPHN) is a major lethal disorder in neonates that leads to an extremely high mortality rate. Thus, the early identification of adverse outcomes in PPHN is critical for clinical practice. This research attempted to develop a nomogram prediction system for assessing the mortality of newborns with PPHN.

**Methods:**

Two hundred and three newborns with PPHN diagnosed from January 2015 to March 2022 were involved in the study. The clinical features of these newborns and pregnancy details were compared between newborns in the survival and lethal groups. Univariable and multivariate analyses were established in sequence to demonstrate the essential risk factors. The nomogram prediction model was built.

**Results:**

A total of 203 newborns were included in the analysis. 136 (67.0%) newborns represented the hospital survival group. Plasma pH value (OR = 0.606, *p* = 0.000, 95% CI 0.45715–0.80315), septicemia (OR = 3.544, *p* = 0.000, 95% CI 1.85160–6.78300), and abnormal pregnancy history (OR = 3.331, *p* = 0.008, 95% CI 1.37550–8.06680) were identified as independent risk factors for neonatal death in newborns associated with PPHN. Finally, the nomogram predictive model was established based on multivariate analysis results, indicating the efficacies of prediction and calibration.

**Conclusion:**

This study generated an applicable risk score formula using the plasma pH value, septicemia, and abnormal pregnancy history to recognize neonatal death in newborns with PPHN, presenting a sufficient predictive value and calibration.

## Introduction

Persistent pulmonary hypertension of the newborn (PPHN) is a disease that occurs during the prenatal to postnatal circulatory transition, leading to a phenotype with elevated pulmonary vascular resistance and hypoxemia. According to previous research, the mortality rate of PPHN is significantly high (approximately 33%) in the perinatal phase. In the past, studies have been conducted to reduce the lethal rate associated with PPHN. Such studies predicted that the mortality rate could decrease by 7–15% in developed high-income countries. However, an extensive mortality rate distribution (12–40%) exists in lower-income regions (1–4). Given the high mortality rate, identifying efficient risk factors for mortality is essential to manage the clinical practice of PPHN.

In clinical practice, it is urgently necessary to establish a helpful tool for predicting adverse outcomes or early mortality of newborns with PPHN, leading to personalized management based on risk stratification. Several studies have identified risk factors in predicting early death in neonates with PPHN, including an exclusive right-to-left ductal shunt, non-response to inhaled nitric oxide (iNO), 1-min Apgar score ≤3 points, infants with congenital anomalies of the respiratory tract, gestational age <34 weeks, neonates with pneumothorax and acute kidney injury, lung hypoplasia (LH), cesarean section mode of delivery, congenital diaphragmatic hernia (CDH), treatment with high-frequency oscillation ventilation (HFOV), and a decreased pH value (1–3, 5–7). However, the risk factors derived from different studies are controversial, and a more applicable and efficient predictive model needs to be established. Encouragingly, the nomogram prediction score, which incorporates the disease characteristics of an individual instead of a single indicator, has been widely identified as a helpful tool in predicting the prognosis of different diseases in clinical practice (8, 9). The nomogram system has demonstrated higher efficiency in assessing several conditions’ prognoses than simple models (10, 11). This study aimed to determine the risk factors for early death in newborns with PPHN. Based on the risk factors, we attempted to set up a nomogram prediction model.

## Materials and methods

### Patients

This was a retrospective, single-center, observational study that enrolled neonates with established PPHN who presented with a history of singleton delivery between January 2015 and March 2022 in West China Second University Hospital, Sichuan University. The present study conformed to the principles of the Declaration of Helsinki. The Ethics Committee approved this study of the West China Second Hospital of Sichuan University (2014-034). Two trained individual physicians collected the data. Electronic medical records confirmed all clinical data.

The inclusion criteria were as follows: (1) newborns diagnosed with PPHN according to the ESC/ERS Guidelines for the diagnosis and treatment of pulmonary hypertension (2022) and AHA guidelines of pediatric pulmonary hypertension (2015); (2) clinical manifestations of refractory hypoxemia identified according to one of the following three criteria: (i) pulmonary artery systolic pressure (PASP) >40 mmHg (12); (ii) a difference of preductal and postductal arterial oxygen saturation (SaO_2_) >5 to 10%; (iii) a difference of preductal and postductal partial pressure of oxygen (PaO_2_) >10–20 mmHg; (3) PPHN was diagnosed within the first 28 days after birth; (4) PPHN was confirmed by echocardiography; (5) newborns completed a follow-up minimum of 6 months; (6) enrolled newborns were singleton pregnancies; and (7) the parents or guardians of the newborns signed the agreement form to be involved in the research. The exclusion criteria included the following: (1) newborn had a suspected cardiomyopathy; (2) newborns had severe structural malformations of the pulmonary veins; (3) newborns suffered from neonatal or fetal neoplasm; (4) suspected or confirmed chromosomal abnormality; (5) lethal coagulopathy or severe bleeding; (6) positive family history of pulmonary hypertension or suspected inherited pulmonary hypertension; and (7) medical records were incomplete.

### Risk factor analysis

The newborns’ demographic data, maternal pregnancy history, and delivery conditions were documented. Echocardiography and blood tests were conducted and analyzed. Four categories of data were collected that contained the variables considered in our modeling: demographics [sex, birth weight, pregnancy complications, maternal age, abnormal pregnancy-labor history, *in vitro* fertilization and embryo transfer (IVF-ET), gestational age, Apgar score, cesarean or natural birth]; echocardiography [right atrium, right ventricle, left atrium, left ventricle, systolic pulmonary arteria pressure, fractional shortening (FS), ejection fraction (EF), size of patent ductus arteriosus (PDA)]; associated diseases [CDH, pneumonia, neonatal respiratory distress syndrome (NRDS), bronchopulmonary dysplasia (BPD), pneumothorax, asphyxia, hypoxic ischemic encephalopathy (HIE), neonatal meconium aspiration syndrome (MAS), acute kidney injury (AKI), septicemia, intracranial hemorrhage]; and blood test results [complete blood count, liver function, blood electrolyte, renal function, arterial blood gas analysis, coagulation function test, cardiac troponin I (I), creatine kinase-MB (CK-MB), brain natriuretic peptide (BNP)].

### Model construction and evaluation

This study summarized the demographic and clinical characteristics of the patients as continuous and categorical variables. Early death in PPHN was identified as any lethality within the first 6 months postnatally. Associations of the risk of early death in newborns with PPHN with risk factors were evaluated by univariable analysis. Multivariable analysis was then performed using logistic regression to identify independent factors among the significant results found in the univariable analysis. Nomogram prediction scores were formulated based on the results of independent risk factors according to the multivariable analyses. Stepwise regression was used to identify the indicators for inclusion in the nomogram. The receiver operating characteristic curve (ROC) with the area under the ROC curve (AUC) values was used to evaluate the effects of the prediction. Calibration of the prediction model was established by a visual calibration plot comparing the predicted and actual probability of survival in newborns with PPHN to identify those at risk of early death.

### Statistical analysis

Univariable analysis was conducted using IBM SPSS 26.0 (IBM SPSS Inc. Chicago, IL, USA). Quantitative data are presented as the means and standard deviations (SDs), while qualitative data are expressed as numbers of individuals. Differences between the two groups were assessed using independent *t*-tests or Mann–Whitney *U*-tests for continuous variables and the χ2 test or Fisher’s exact test for categorical variables. Multivariable logistic regression analyses, nomograms, and model evaluation were formulated and conducted using R (version 4.1.2) within the RStudio platform. *P*-values < 0.05 were considered statistically significant.

## Results

### Study population

**FIGURE 1:**
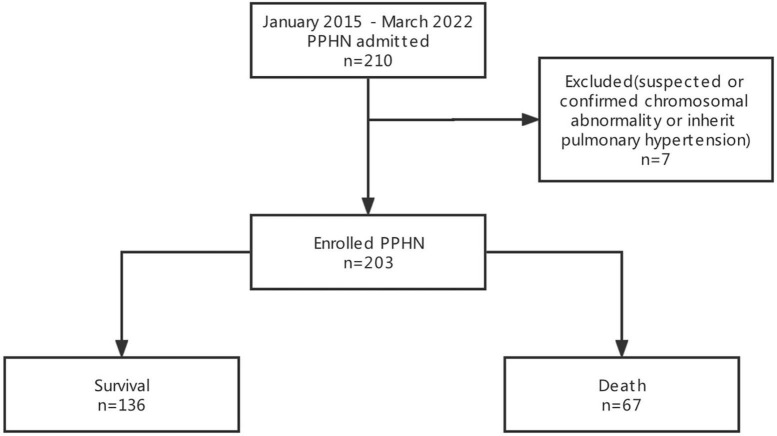
A flow diagram of study participants.

A total of 220 newborns matched the inclusion criteria. Seven newborns with suspected or confirmed chromosomal abnormalities or inherited pulmonary hypertension were excluded. Thus, 203 newborns were included to develop the nomogram prediction model. The baseline information of the participants is summarized in Table 1. Briefly, 67 out of 203 newborns (37%) suffered early death associated with PPHN (Figure 1). Furthermore, all the first-degree family members of the involved patients were absent from the history of PPHN.

**TABLE 1:** Univariate analysis for risk factors.

Variables	Survival (*n* = 136)	Death (*n* = 67)	*P*-value
Male gender, *n* (%)	72 (52.94%)	29 (43.28%)	0.196
Birth weight, *n* (%)			0.008
<1000 g	9 (6.62%)	9 (13.43%)	
<1500 g	11 (8.09%)	6 (8.96%)	
<2500 g	21 (15.44%)	17 (25.37%)	
≥ 2500 and ≤ 4000 g	93 (68.38%)	34 (40.75%)	
>4000 g	2 (1.47%)	1 (1.49%)	
Abnormal pregnancy- labor history, *n* (%)	12 (8.82%)	16 (23.88%)	0.003
Cesarean, *n* (%)	93 (68.38%)	38 (56.72%)	0.102
IVF-ET, *n* (%)	13 (9.56%)	13 (19.40%)	0.048
Gestational age, *n* (%)			0.144
≥37 weeks	83 (61.03%)	31 (46.27%)	
32–36 + 6	33 (24.26%)	18 (26.67%)	
28–31 + 6	10 (7.35%)	9 (13.43%)	
≤28 weeks	10 (7.35%)	9 (13.43%)	
**Complications of puerpera**			
Hypertensive disorders of pregnancy, *n* (%)	8 (5.88%)	4 (5.97%)	1.000
Gestational diabetes mellitus, *n* (%)	37 (27.21%)	20 (29.85%)	0.693
ICP, *n* (%)	9 (6.62%)	2 (3.00%)	0.456
Premature rupture of membranes, *n* (%)	32 (23.53%)	14 (20.90%)	0.673
**Complications of newborn**			
Diaphragmatic hernia, *n* (%)	4 (2.94%)	4 (5.97%)	0.510
Asphyxia, *n* (%)	45 (33.09%)	33 (49.25%)	0.026
MAS, *n* (%)	16 (11.76%)	7 (10.45%)	0.781
Pneumonia, *n* (%)	112 (82.35%)	53 (79.10%)	0.577
NRDS, *n* (%)	68 (50%)	40 (59.70%)	0.193
BPD, *n* (%)	17 (12.50%)	7 (10.45%)	0.670
Air leak of the newborn, *n* (%)	18 (13.24%)	9 (13.43%)	0.969
Septicemia, *n* (%)	43 (31.62%)	42 (62.69%)	0.000
HIE, *n* (%)	5 (3.68%)	3 (4.48%)	1.000
Intracranial hemorrhage, *n* (%)	33 (24.26%)	31 (46.27%)	0.002
AKI, *n* (%)	29 (21.32%)	24 (35.82%)	0.027
CHD*, *n* (%)	40 (29.41%)	15 (22.39%)	0.290
**Treatment**			
iNO, *n* (%)	89 (65.44%)	53 (79.10%)	0.046
PS, *n* (%)	67 (49.26%)	34 (50.75%)	0.843
Sildenafil, *n* (%)	13 (9.56%)	9 (13.43%)	0.404
Dopamine, *n* (%)	60 (44.12%)	42 (62.69%)	0.013
Alprostadil, *n* (%)	16 (11.76%)	15 (22.39%)	0.048
Noradrenalin, *n* (%)	20 (14.71%)	20 (29.85%)	0.011
Milrinone, *n* (%)	31 (22.79%)	16 (23.88%)	0.771
**Echocardiography**			
Mitral reflux, *n* (%)	29 (21.32%)	12 (17.91%)	0.569
PDA reflux, *n* (%)	43 (31.62%)	23 (34.33%)	0.698
**Complete blood count**			
White blood cell count (WBC × 10^9/L)	15.31 ± 8.12	16.86 ± 10.03	0.255
Neutrophil (× 10^9/L)	11.13 ± 7.94	12.51 ± 8.21	0.199
Lymphocyte (× 10^9/L)	3.92 ± 3.17	5.42 ± 6.28	0.465
Red blood cell count (RBC × 10^9/L)	4.43 ± 0.82	4.05 ± 1.06	0.013
Hemoglobin (HGB, g/L)	157.05 ± 30.91	144.63 ± 37.95	0.013
Red cell distribution width (RDW-SD, fl)	63.11 ± 10.17	65.80 ± 8.08	0.007
Hematocrit (HCT, %)	45.92 ± 9.70	43.66 ± 10.72	0.133
Platelet count (PLT × 10^9/L)	237.21 ± 88.38	206.87 ± 92.48	0.025
Platelet distribution width (PDW, fL)	11.55 ± 2.35	12.08 ± 2.79	0.154
Platelet-larger cell ratio (P-LCR, %)	26.97 ± 6.96	29.30 ± 7.44	0.020
Mean platelet volume (MPV, fL)	10.36 ± 0.91	10.68 ± 0.98	0.016
C-reactive protein (CRP, mg/L)	5.90 ± 11.60	10.14 ± 20.06	0.124
**Liver function test**			
Alanine aminotransferase (ALT, U/L)	23.62 ± 25.62	87.57 ± 371.40	0.539
Aspartate aminotransferase (AST, U/L)	83.25 ± 67.33	404.34 ± 1579.11	0.121
Total bilirubin (TB, μmol/L)	98.02 ± 67.45	95.13 ± 79.23	0.451
Direct bilirubin (DBIL, μmol/L)	11.83 ± 27.58	9.47 ± 7.29	0.642
Indirect bilirubin (IDIL, μmol/L)	86.55 ± 60.28	85.67 ± 75.83	0.474
γ-glutamyl transpeptidase (γGT, U/L)	157.74 ± 105.19	189.19 ± 156.38	0.139
Lactate dehydrogenase (LDH, U/L)	1096.17 ± 929.26	1769.54 ± 2627.72	0.075
Albumin (ALB, g/L)	30.65 ± 5.12	28.99 ± 6.48	0.071
Globulin (GLB, g/L)	17.92 ± 4.67	16.39 ± 5.11	0.035
**Kidney function test**			
Urea (UN, mmol/L)	4.98 ± 2.88	12.65 ± 55.02	0.120
Creatinine (Cr, μmol/L)	62.54 ± 54.92	64.63 ± 26.11	0.056
**Blood electrolyte**			
Potassium (K +, mmol/L)	4.43 ± 0.82	4.67 ± 1.15	0.392
Calcium (Ca_2_ +, mmol/L)	2.05 ± 0.36	1.93 ± 0.41	0.038
Chloride (Cl-, mmol/L)	104.98 ± 4.60	103.19 ± 12.65	0.578
Sodium (Na +, mmol/L)	138.55 ± 3.69	137.30 ± 5.33	0.088
**Arterial blood gas analysis**			
plasma pH	7.35 ± 0.11	7.27 ± 0.19	0.005
PaO_2_ (mmHg)	66.34 ± 16.18	62.06 ± 16.46	0.079
PaCO_2_ (mmHg)	39.71 ± 9.93	41.90 ± 14.67	0.291
**Echocardiography**			
FS (%)	33.38 ± 4.58	33.27 ± 5.18	0.659
EF (%)	64.55 ± 6.55	64.34 ± 6.70	0.704
Size of PDA (mm)	2.13 ± 1.52	2.40 ± 1.65	0.491
**Treatment**			
Duration of nitric oxide (day)	3.04 ± 3.54	2.22 ± 2.47	0.257
Dose of nitric oxide (ppm)	14.30 ± 12.70	19.79 ± 14.36	0.012
Duration of milrinone (day)	0.93 ± 2.96	0.63 ± 1.55	0.896
Dose of milrinone (ug/kg/min)	0.09 ± 0.18	0.09 ± 0.21	0.903
			

ICP, intrahepatic cholestasis of pregnancy.

*CHD include atrial septal defects, ventricular septal defects, pulmonic stenosis, aortic stenosis, coarctation of the aorta, bicuspid aortic valve stenosis, subaortic stenosis, persistent truncus arteriosus, pulmonary venous connection, tetralogy of Fallot, hypoplastic left heart syndrome, hypoplastic right heart syndrome, Mitral stenosis, transposition of the great vessels, tricuspid atresia, Wolff–Parkinson-white syndrome, double aortic arch, aberrant subclavian artery, interrupted aortic arch and scimitar syndrome.

### Univariate analyses between the survival group and death group

As shown in Table 1, the univariable analysis revealed several parameters associated with earth death in newborns with PPHN, including abnormal pregnancy history (*P* = 0.03), IVF-ET (*P* = 0.048), birth weight (*P* = 0.008), history of asphyxia (*P* = 0.026), septicemia (*P* = 0.000), intracranial hemorrhage (*P* = 0.002), acute kidney injury (*P* = 0.027), application of iNO (*P* = 0.046), high-dose nitric oxide administration (*P* = 0.012), dopamine application (*P* = 0.013), alprostadil administration (*P* = 0.048), treatment with noradrenaline (*P* = 0.011), red blood cell count (*P* = 0.013), mean platelet volume (*P* = 0.016), platelet-larger cell ratio (*P* = 0.020), serum calcium (*P* = 0.038), platelets (*P* = 0.025), globulin (*P* = 0.035), plasma pH (*P* = 0.005), and red cell distribution width (*P* = 0.007).

### Selected factors for the prediction model

After univariable analysis, the variables of abnormal pregnancy history, IVF-E, birth weight, history of asphyxia, septicemia, intracranial hemorrhage, AKI, application of iNO, high-dose nitric oxide administration, dopamine application, alprostadil administration, treatment with noradrenaline, red blood cell count, mean platelet volume, platelet-larger cell ratio, serum calcium, platelet, globulin, plasma pH and red cell distribution width were entered into the multivariate logistic regression analysis. Multivariable logistic regression analysis identified pH (OR = 0.606, *P* = 0.000, 95% CI 0.457–0.803), septicemia (OR = 3.544, *P* = 0.000, 95% CI 1.851–6.783), and abnormal pregnancy history (OR = 3.331, *P* = 0.008, 95% CI 1.376–8.067) as independent predictors of survival in newborns with PPHN (Table 2).

**TABLE 2:** Multivariable analysis for risk factors for PPHN-associated early death.

	Estimate	Std. error	*z*-value	Pr (>|z|)
Abnormal pregnancy-labor history	1.187	5.357	2.216	0.02667
Septicemia	1.015	4.424	2.295	0.02171
Plasma pH	–4.437	1.398	–3.173	0.00151
IVE-ET	9.482	5.670	1.672	0.09446
Asphyxia	–3.544	4.700	0.000	0.99994
Intracranial hemorrhage	6.085	4.135	1.472	0.14114
AKI	–9.073	4.271	–0.212	0.83178
iNO	–6.479	6.833	–0.948	0.34304
Dose of nitric oxide	1.688	2.267	0.745	0.45654
Treatment with dopamine	–5.963	4.693	–1.271	0.20384
Treatment with alprostadil	9.710	5.239	1.854	0.06379
Treatment with noradrenalin	5.040	4.728	1.066	0.28642
RBC	–4.103	5.246	–0.078	0.93766
MPV	1.039	1.103	0.942	0.34632
P-LCR	–9.864	1.464	–0.701	0.50038
Ca_2_ +	–8.129	5.334	–1.524	0.12750
PLT	6.322	2.202	0.287	0.77400
GLB	–2.921	4.168	–0.701	0.48334
Birth weight	–4.211	1.909	–0.221	0.82542
RDW-SD	9.905	2.093	0.473	0.63611
				

### Development of an individualized prediction nomogram

**FIGURE 2:**
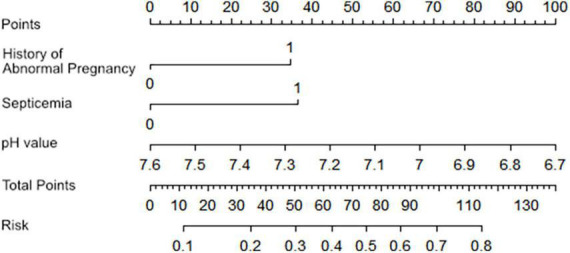
The nomogram prediction scores of multivariable analysis. The value of each variable was given a score on the point scale axis. A total score could be easily calculated by adding each single score, and by projecting the total score to the lower total point scale, we were able to estimate the probability of early death among newborns with PPHN.

Based on the multivariable logistic regression analysis, a nomogram prediction model was constructed, which was formulated with three independent risk factors to identify early death in newborns with PPHN (Figure 2). A total score was calculated using the plasma pH, septicemia incidence, and abnormal pregnancy history. Among these variables, plasma pH represented a continuous variable; the other two parameters were binary. The value of each of the involved variables was converted into a score on the point scale axis. The total score could then be calculated by adding every single score together and, by projecting the total score to the estimated risk, allowed us to determine the probability of early death among newborns with PPHN.

### Performance of the nomogram prediction

**FIGURE 3:**
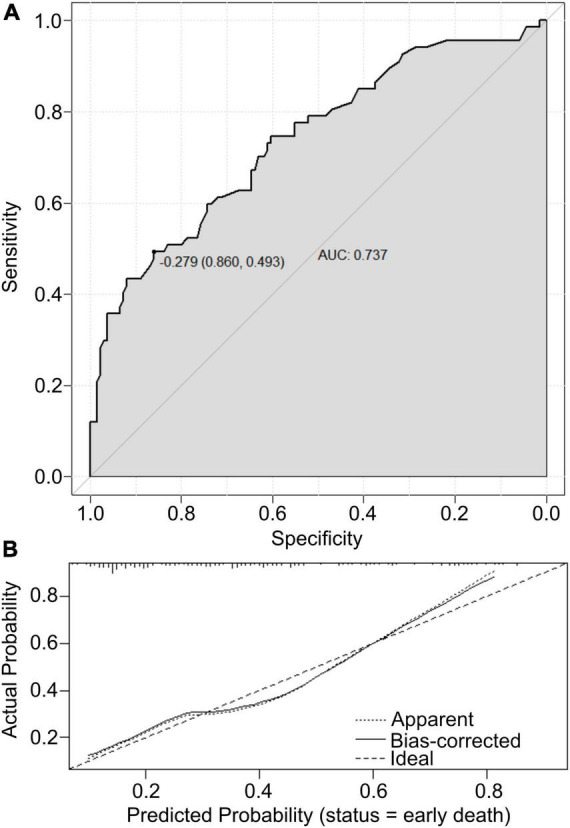
**(A)** The ROC curves of the nomogram scores. **(B)** Calibration plot analyses for nomogram scores.

The value of the established nomogram prediction was measured by predictive ability and calibration. AUC assessed the predictive ability of the nomogram model. The calibration of the established model was evaluated by a visual calibration plot comparing the predicted and actual survival rates of newborns with PPHN. Based on the ROC analysis, the nomogram prediction model presented an efficient prediction, with an AUC of 0.737 (95% CI 0.663–0.812) (Figure 3A). The calibration curve of the nomogram prediction is presented in Figure 3B and reveals that the early death prediction by the nomogram agrees well with the actual mortality rate. The Hosmer–Lemeshow test yielded no statistically significant difference (*P* = 0.636).

## Discussion

The sustained elevation of PVR and pulmonary vessel remodeling characterize PPHN. PPHN represents a significant challenge in managing neonates due to the high rate of adverse outcomes. Lung recruitment, iNO, intratracheal exogenous pulmonary surfactant (PS) therapy, and gentle ventilation strategies would improve the outcomes of PPHN, which would also help to reduce the reliance on extracorporeal membrane oxygenation (ECMO). However, it has been identified that the early or prompt administration of ECMO might increase the survival rate of PPHN. In the present study, a prediction model for early death was developed and evaluated in newborns with PPHN. This nomogram model incorporated the plasma pH value, identification of septicemia, and abnormal pregnancy-labor history and showed an efficient predictive value (AUC = 0.737, 95% CI: 0.663–0.812) and calibration performance. Thus, this nomogram prediction score system was able to facilitate individualized prediction of early death in newborns with PPHN. As such, this nomogram may have great potential in clinical application.

Persistent pulmonary hypertension of the newborn may be primary or secondary to conditions such as pulmonary hypoplasia, NRDS, MAS, CDH, pneumonia, asphyxia, sepsis, or cardiac disorders (5, 13). In animal and human studies, sepsis has been reported as an independent risk factor for PPHN (5, 14). Sepsis complicated by PPHN can lead to severe hypoxemia and worsen the outcomes and course of affected newborns. Several cases of neonatal sepsis-induced PPHN have demonstrated high morbidity and mortality in newborns (15–17). Early detection of PPHN with sepsis may lead to specific interventions associated with more favorable outcomes (18). Severe infection may cause pulmonary vasoconstriction, leading to hypoxia and PPHN (18). Mean PASP was significantly higher in neonates with sepsis than those without infection or other known risk factors for PPHN (19). Previous studies have found that group B Streptococcus (GBS) infection contributes to early onset sepsis in neonates. At the same time, phosphatidylglycerol and cardiolipin, which GBS produces, are considered to be associated with PPHN (20, 21) due to their ability to induce pulmonary hypertensive and pulmonary endothelial injuries. Thus, early administration of penicillin after birth may help to reduce the severity of PPHN associated with GBS infection. However, penicillin treatment of Streptococci also induces an immediate secretion of phospholipids, which may contribute to developing PPHN (22). Furthermore, long-term usage of penicillin increases lipid synthesis. Thus, the timing and duration of antibiotic application are critical in managing PPHN in newborns (14).

This study identified a history of abnormal pregnancy as an independent risk factor for early death associated with PPHN. An abnormal pregnancy-labor history was related to genetic abnormalities and harmful environmental exposures. Although the present study excluded cases of chromosomal diseases and inherited cardiovascular diseases, some newborns may have suffered from genetic malformations that contributed to adverse outcomes, as the subjects of this study were not required to undergo genetic testing. As such, the present study failed to determine whether PPHN was associated with genetic mutations. Alterations in lung development and genetic conditions are essential in developing PPHN. Moreover, several maternal and fetal issues encode the origins of PPHN prenatally. Previous studies have demonstrated a link between genetic polymorphisms of the urea cycle enzyme and the onset of PPHN (23). In addition, transforming growth factor-β (TGF-β) and the endothelin system have been implicated in PPHN (24–26). Impaired expression of vasoactive substances also contributes to progressive changes in pulmonary vasoreactivity, which are tightly related to the variant expression of the *NOS3*, *VEGFA*, and *EDN1* genes (27–30). *EDN1* has been involved in several cardiovascular disorders, including high blood pressure and pulmonary hypertension (31–33). The rs2070699 SNP of *EDN1* has been suspected to be associated with neonatal respiratory distress and PPHN. In addition, the *ABCA3* gene has also been identified to contribute to neonatal recurrent pulmonary hypertension and respiratory failure (34). Accordingly, prenatal genetic screens are critical for newborns with highly suspected PPHN. These screens may also be beneficial for the subsequent pregnancy. However, genetic variance contributes to identifying the risk factor of PPHN. Nevertheless, the application of genetic tests was expensive and led to an extended analysis duration. So, we only enrolled genetic tests in the highly suspected syndrome patients. As all the PPHN patients with syndromes were excluded, the general patients did not receive any genetic tests in this study.

Responses to iNO are also correlated with the severity of PPHN and facilitate optimal lung aeration by intratracheal exogenous PS. However, evidence has demonstrated that a large number of infants fail to respond to the administration of iNO. The plasma pH value is critical to predict the responses of iNO treatment, and the normal range of pH values between 7.40 and 7.45 is more likely to correspond to iNO treatment. Previous studies have shown that a PaO_2_/FiO_2_ < 50 and a pH value < 7.2 indicate more severe PPHN and a higher probability of early death. The degree of acidosis forms a negative feedback loop with pulmonary hypertension, resulting in poor iNO treatment. Morel et al. showed that low PaO_2_ and pH values cause a negative response to iNO treatment (35). Heidersbach et al. revealed that the administration of iNO, alkalosis and oxygen presented a dose-dependent effect on pulmonary vasodilation in a lamb model. They revealed that iNO-induced pulmonary vasodilation is efficient when the systemic arterial pH > 7.40 (36, 37). Thus, the arterial pH value was crucial to assess the outcomes of PPHN.

## Limitation

There are some limitations in this study. Selective bias may have occurred, as this study was performed in a single institution. Some of the data in this study are not comprehensive and cannot be included in the multivariate analysis. The ratio of ECMO application was not high enough, which may have affected the survival rate of newborns. This study discusses the genetic variance associated with PPHN, but the subjects of this study did not undergo genetic testing. Genetic data combining clinical characteristics may improve the prediction model performance.

## Conclusion

Persistent pulmonary hypertension of the newborn is a life-threatening condition that reveals significant challenges in neonatal management, leading to a high morbidity and mortality rate. An advanced predictive system would help to improve the prognosis among such patients and maintain the therapeutic strategy. This study generated a novel applicable risk score formula using the plasma pH value, septicemia, and abnormal pregnancy history to recognize neonatal death in newborns with PPHN by nomogram prediction method. This score formula presented an efficient predictive value and calibration in PPHN.

## Data availability statement

The original contributions presented in this study are included in the article/supplementary material, further inquiries can be directed to the corresponding authors.

## Ethics statement

This study was approved by the Ethics Committee of West China Second Hospital of Sichuan University (2014-034). Written informed consent to participate in this study was provided by the participants’ legal guardian/next of kin.

## Author contributions

CL, JM, YZ, SD, and JW collected the clinical data. CL and JM reviewed the literature, contributed to the manuscript drafting, and performed the nomogram analysis. JW and YL conceptualized and designed the study, coordinated and supervised data collection, critically reviewed the manuscript for important intellectual content, and responsible for the revision of the manuscript for important intellectual content. All authors issued final approval for the version to be submitted.
